# Adjustment Disorder Presenting as Syncope in a Patient With Wolf-Parkinson White Syndrome

**DOI:** 10.7759/cureus.74046

**Published:** 2024-11-19

**Authors:** Jonathan Van Name

**Affiliations:** 1 Internal Medicine, University of Florida College of Medicine, Gainesville, USA

**Keywords:** adjustment disorder, anxiety, psychosocial stress, syncope, wolf-parkinson-white

## Abstract

Adjustment disorder encompasses maladaptive emotional, behavioral, and physiologic symptomatology that is related to an identifiable psychosocial stressor. Adjustment disorder manifesting as syncope in a patient with Wolf-Parkinson White (WPW) Syndrome is uncommon and has not previously been documented in medical literature. In this case, we discuss a 24-year-old male with a history of WPW who presented with unexplained, episodic syncope in the setting of acute life stressors. A patient with no prior neurologic or psychiatric history was referred to the hospital for more than 15 unexplained syncopal episodes in the preceding week. The patient was confirmed to have WPW based on EKG interpretation, but cardiac work-up, including telemetry, troponins, and orthostatic vital signs, were within normal limits. Additionally, WPW was ruled out as the cause of syncope since there were no cardiac electrophysiologic changes during the syncopal episodes. While in the hospital, the patient had episodic syncopal episodes every three to four hours, with no identified triggers or patterns. A thorough seizure work-up consisting of EEG and neurological consultation was normal. After discussion with psychiatric services, the patient was diagnosed with adjustment disorder in the setting of significant psychosocial stressors following a recent legal arrest. The patient was discharged with escitalopram and was instructed to follow up closely with cardiology for cardiac event monitoring. This case report illustrates the importance of considering psychosocial factors when creating a differential diagnosis for syncope.

## Introduction

Adjustment disorder is a psychological condition that arises in response to identifiable stressors or life changes, manifesting itself with behavioral, emotional, and sometimes neurological symptoms disproportionate to the severity of the stress [1]. According to the American Psychiatric Association's Diagnostic and Statistical Manual of Mental Disorders, Fifth Edition (DSM-5), this symptomatology occurs within three months of the onset of the stressor [2]. Additionally, while the symptoms of adjustment disorder are usually temporary, they may persistently cause functional limitation up to six months after the stressor has been terminated [3]. Adjustment disorder may present similarly to other functional neurologic and somatic symptom disorders. However, a key difference between adjustment disorder and these similar psychological conditions is the presence of emotional distress, including feelings of sadness, excessive worrying, and difficulty concentrating. Conversely, functional neurologic disorders are often associated with la belle indifference, which describes a paradoxical absence of psychological distress despite serious symptoms or illness. 

Recent studies demonstrate that the prevalence of adjustment disorder is approximately 11.5% and is most common in the 15-25 years of age group [4]. While adjustment disorder is typically described by emotional distress (anxiety, depression, etc.) or maladaptive behavioral changes (poor concentration, insomnia, etc.), it can also present with somatic complaints, headaches, and syncope. A psychosomatic characterization study demonstrated an overlap in patients with functional somatic and conversion symptoms in nearly 37% of the adjustment disorder population [5]. It is posited that sympathetic stress responses play a significant role in this neurological symptomatology. Specifically, patients with adjustment disorder are more likely to experience sympathetic dysfunction, which may present as a recurrent vasovagal syncope phenotype. Given the overlap of neurologic symptoms between adjustment disorder and functional neurogenic etiologies, diagnosing patients with predominantly physical symptoms can be challenging. Thus, it is important to factor in the timing of psychosocial stressors and the presence of emotional and behavioral symptomatology in these cases.

While adjustment disorder can occur in individuals of all ages, younger individuals who have a history of mental health illness are most susceptible. Early identification and treatment are paramount when managing the disorder, as psychotherapeutic techniques and pharmacologic measures can alleviate the acute symptoms associated with adjustment disorder. Based on the literature review, nearly 97% of patients recover from adjustment disorder without long-term complications [6]. Although research is limited regarding combined psychotherapy (cognitive behavioral therapy based) and pharmacology, it has been shown that combined therapy may lead to a reduction in psychopathological symptoms post-treatment [7]. Given the diagnostic challenges of adjustment disorder, patients with adjustment disorder may be at higher risk of developing major depressive disorder in the future.

This case report also discusses WPW syndrome, a congenital cardiac pre-excitation syndrome that arises from abnormal cardiac electrical conduction through an accessory pathway. Patients with WPW syndrome can experience a wide range of symptomatology, including tachycardia, palpitations, episodic lightheadedness, syncope, and rarely cardiac arrest. In addition to these symptoms, WPW syndrome can lead to malignant tachyarrhythmias including atrial fibrillation, paroxysmal supraventricular tachycardias, and ventricular fibrillation. Failure to carefully evaluate WPW-related syncope can lead to potentially fatal sequelae including cardiac arrest. Thus, this case report illustrates the importance of thorough cardiac work-up when determining syncopal etiologies.

## Case presentation

A 24-year-old Caucasian male in the United States with a history of WPW syndrome presented to the hospital with recurrent syncopal episodes in the preceding seven days. On admission, he was hemodynamically stable, with vitals: Temp 36.7 ºC, heart rate (HR) 80, blood pressure (BP) 120/71, respiratory rate (RR) 17, O_2_ 96% on room air. He endorsed having more than 15 episodes described as “sudden loss of consciousness” in the past week, which began two weeks after having an arrest for multiple behavioral disruptions. The patient was not able to describe a specific frequency or pattern for syncopal episodes and reported that they occurred both at rest and during activity. Given his history of WPW, he was initially concerned that his syncope was arrhythmic in etiology, so he presented to an outside hospital for initial evaluation. At the outside hospital, an EEG and brain MRI were within normal limits, and neurological causes were ruled out. The patient was transferred for further cardiac and psychiatric evaluation.

Upon arrival at our emergency department, the patient had a witnessed syncopal event in his hospital bed. This event was described as sudden loss of consciousness lasting ten to fifteen seconds, with no changes in vitals or cardiac rhythm evident on telemetric monitoring. He spontaneously regained consciousness with a brief period of confusion lasting less than one minute. During this episode, no rhythmic movements or other seizure characteristics were identified.

**Figure 1:**
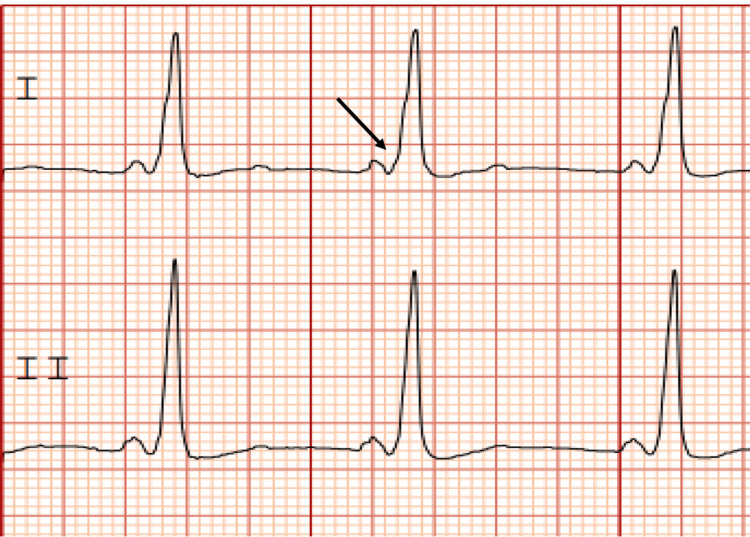
The patient's initial EKG Pre-excitation (i.e., delta wave) is shown in leads I and II on the patient's initial electrocardiogram (EKG). The arrow demonstrates the characteristic delta wave as evidence of pre-excitation in Wolf-Parkinson White (WPW) syndrome. This EKG demonstrates that the patient is in normal sinus rhythm at baseline.

After this initial episode, further work-up at the hospital consisted of a CT head without contrast, which was negative for intracranial abnormality. An EKG demonstrated pre-excitation (evidence of delta-waves as shown in Figure 1), confirming his diagnosis of WPW Syndrome, with no concerns for additional arrhythmia. A transthoracic echocardiogram showed a normal ejection fraction with no structural changes, indicating no cardiac wall damage or obstructive hypertrophy. The patient’s vitals remained stable throughout hospitalization, and the patient demonstrated no signs of orthostasis or autonomic instability. Laboratory tests were notable for no anemia or electrolyte abnormalities. High-sensitivity troponins were negative. Given the patient’s emotional changes from baseline and new-onset neurological symptoms, a brief work-up was ordered to rule out infectious etiologies. His C-reactive protein level (CRP) was mildly elevated at 6.51 mg/L (normal range: 0.00-5.00 mg/L); however, syphilis screening and mononucleosis testing were both negative.

**Figure 2:**
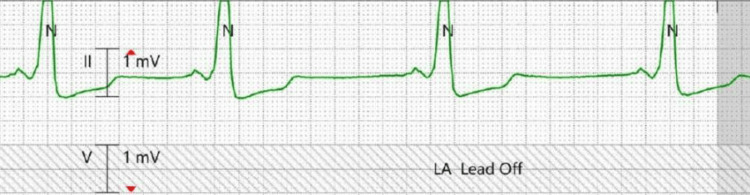
Telemetric data collected during syncopal event Telemetry strip showing no electrocardiographic changes compared to baseline during syncopal event. This telemetry strip demonstrates that the patient is in normal sinus rhythm during syncope.

During the hospital course, consultation from cardiology, neurology, and psychiatry was requested. Based on telemetric data showing no electrophysiological changes during recurrent syncopal episodes, there was low concern that WPW was the cause of this patient’s syncope (Figure 2). Additionally, given no urinary/bowel incontinence or tongue biting with no postictal period, neurology had low suspicion for epileptic events. Based on psychiatry’s assessment, the patient was diagnosed with adjustment disorder with depressed and anxious mood in the setting of significant psychosocial stressors (i.e., recent legal arrest) occurring less than one month prior to syncopal onset. The patient met DSM-5 criteria for adjustment disorder, given the development of behavioral symptoms in response to an identifiable stressor within three months of stressor onset, in addition to marked distress out of proportion to the severity of the stressor and significant functional impairment.

Based on both depression and some mild anxiety symptoms, escitalopram 10 mg once daily was started per psychiatry while in-patient, and the patient was instructed to remain on this medication for at least six months. Additionally, a referral to outpatient psychiatry was ordered, with an emphasis on cognitive behavioral therapy, stress-reduction management, and medication management. It was discussed with the patient that failure to follow closely with psychiatry could yield prolonged behavioral and neurological symptoms. The patient was instructed to follow up with cardiology for outpatient cardiac event monitoring. He was advised to monitor for increasing syncopal episode frequency, increasing duration of syncopal episodes, and worsening tachycardia or palpitations in the interim.

## Discussion

In this case, a patient with known WPW syndrome was found to have recurrent syncope as a result of recent life stressors. As mentioned previously, adjustment disorder is a psychological condition that arises in response to identifiable stressors. As is evidenced in recent literature reviews, there is a profound link between cardiovascular and mental health, emphasizing the importance of a holistic approach that integrates the treatment of physical symptoms while addressing mental health issues [8]. The symptoms of adjustment disorder vary significantly, and as described, can include intense somatic symptoms, such as headaches, gastrointestinal disturbances, and syncope [9]. Autonomic dysregulation occurring as a result of a dysregulated sympathetic stress response may present as syncope or near-syncope [10]. This impaired stress response has been shown to cause decreased vagal function, creating a vasovagal neurologic phenotype. This case report demonstrates that prior cardiac arrhythmia history should not preclude additional syncopal work-up or a diagnosis of psychogenic syncope if neurocardiogenic etiologies are cautiously ruled out.

When syncope is reported in the context of a known history of arrhythmia, a comprehensive work-up is essential to rule out other underlying conditions that may contribute to these symptoms. To rule out arrhythmic etiologies, a thorough work-up consisting of telemetric monitoring and EKG is ascertained. To rule out orthostatic and positional hypotension, orthostatic vital signs are important to assess. Additionally, neurological imaging should be ordered to determine if structural or vascular causes of syncope are present. A psychogenic cause should only be considered after these thorough cardiac and neurological evaluations are completed [11]. Additional studies consisting of tilt testing, transcranial Doppler, and epilepsy monitoring are particularly important if initial evaluations are inconclusive or if syncope continues despite non-psychogenic findings [12].

This case also demonstrates the importance of a thorough collection of past medical and social history. In this patient, evidence of a significant life stressor (i.e., recent legal arrest due to unspecified behavioral disruptions) was necessary to clinically diagnose adjustment disorder and to determine the syncopal etiology. Numerous publications, in addition to this case presentation, highlight the importance of in-depth social histories, particularly in the context of uncertain pathologic etiologies [13-15].

Determining the etiology of recurrent syncope is paramount in optimizing management for symptom resolution. In adjustment disorder, treatment is multimodal and consists of cognitive behavioral therapy, stress reduction management, and targeted pharmacotherapy [16]. Early detection and treatment of adjustment disorder is critically important in decreasing the length and severity of symptoms. Cognitive behavioral therapy (CBT) is of particular utility when treating patients with adjustment disorders. For these patients, CBT psychotherapeutic work-up typically consists of thought diaries and addressing negative cognition and automatic thoughts. Specifically, cognitive behavioral therapy allows patients to develop better coping mechanisms and behavioral modifications when exposed to triggering emotional thoughts and stressors. This reduction in emotional response to psychosocial stressors can decrease sympathetic dysfunction and may help reduce recurrent syncope in this patient population. With adequate treatment, patients with adjustment disorder should expect resolution of behavioral and physiologic symptoms within six months from the termination of the stressor. Thus, eliminating the offending psychosocial stressor is paramount for a complete recovery. The multimodal treatments for adjustment disorder are shown in Table 1.

**Table 1:** Multimodal treatment options for adjustment disorder This table describes multimodal options for the treatment of adjustment disorder, including pharmacologic, psychotherapeutic, and behavioral modification strategies. SSRI: Selective serotonin reuptake inhibitor; SNRI: Serotonin–norepinephrine reuptake inhibitors.

Pharmacologic	Clinical Psychotherapy	Behavioral Modifications
SSRIs	Cognitive behavioral therapy	Body-mind-spirit technique
Benzodiazepines	Short-term dynamic psychotherapy	Mindfulness exercises
SNRIs	Humor training	Yoga meditation
Plant extracts (Kava-kava, Ginkgo bilboa)	Bibliotherapy	Mirror therapy

## Conclusions

This case report underscores the importance of avoiding cognitive biases when undergoing syncopal evaluation. Given this patient’s history of WPW, the recurrent syncope could have erroneously been attributed to his known cardiac history. Furthermore, this case report demonstrates the importance of interdisciplinary collaboration when determining the correct diagnosis for syncope of unknown etiology. Specifically, psychiatry was essential in psychosocial assessment, neurology helped rule out seizures, and the cardiology service was needed for arrhythmia evaluation. Additionally, this case report highlights the necessity of collecting thorough past medical and social histories, and how psychosocial context influences treatment decisions and improves overall patient outcomes. This report also demonstrates the need for complete evaluation with laboratory and imaging evidence to avoid prematurely attributing symptoms to an existing diagnosis. When effectively diagnosed and treated, patients with adjustment disorder should expect complete symptom resolution once psychosocial stressors are adequately controlled. 
